# Cardiovascular and respiratory emergency dispatch due to short-term exposure to ambient PM10 in Dezful, Iran

**DOI:** 10.15171/jcvtr.2019.44

**Published:** 2019-10-13

**Authors:** Hamidreza Aghababaeian, Maryam Dastoorpoor, Afsaneh Ghasemi, Maryam Kiarsi, Narges Khanjani, Ladan Araghi Ahvazi

**Affiliations:** ^1^Nursing and Emergency Department, Dezful University of Medical Sciences, Dezful, Iran; ^2^Department of Health in Emergencies and Disaster, School of Public Health, Tehran University of Medical Sciences, Tehran, Iran; ^3^Department of Biostatistics and Epidemiology, Menopause Andropause Research Center, Ahvaz Jundishapur University of Medical Sciences, Ahvaz, Iran; ^4^Air Pollution and Respiratory Diseases Research Center, Ahvaz Jundishapur University of Medical Sciences, Ahvaz, Iran; ^5^Department of Public Health, School of Public Health, Fasa University of Medical Sciences, Fasa, Iran; ^6^Environmental Health Engineering Research Center, Kerman University of Medical Sciences, Kerman, Iran

**Keywords:** Accidents, Cardiovascular System, Emergency Medical Services, Particulate Matter, Respiratory System

## Abstract

***Introduction: *** This study was conducted to determine the relation between exposure to particulate matter less than 10 microns (PM_10_) caused by dust storms and the risk of cardiovascular, respiratory and traffic accident missions carried out by Emergency Medical Services (EMS).

***Methods:*** This was a time-series study conducted in Dezful city, Iran. Daily information on the number of missions by the EMS due to cardiovascular, respiratory and crash problems and data on PM_10_ were inquired from March 2013 until March 2016. A generalized linear model (GLM) with distributed lag models (DLMs) was used to evaluate the relation between the number of EMS missions and the average daily PM_10_. The latent effects of PM_10_ were estimated in single and cumulative lags, up to 14 days.

***Results:*** In the adjusted model, for each IQR increase in the average daily PM_10_ concentration, the risk of EMS missions in the total population in single lags of 2 to 7 days, and the cumulative lags of 0-7 and 0-14 days after exposure had a 0.8, 0.8, 0.8, 0.8, 0.7, 0.6, 6.7 and 1.4% significant increase. Also, for each IQR increase in the daily mean concentration of PM_10_ in single 1 to 7, and cumulative lags of 0-2, 0-7, and 0-14 days after exposure, respectively, a 2.4, 2.7, 2.8, 2.9, 2.9, 2.7, 2.5, 7.4, 23.5 and 33. 3 % increase was observed in the risk of EMS cardiovascular missions.

***Conclusion:*** Increase in daily PM_10_ concentrations in Dezful is associated with an increase in the risk of EMS missions in lags up to two weeks after exposure.

## Introduction

Environmental pollutants are seriously endangering the 21st century ecosystems, and among them, air pollution is of particular importance.^1^ In fact, air pollution is one of the most important health concerns due to its adverse effects on human health.^2^ The WHO estimated that as many as 7 million people died in 2012 because of exposure to polluted air.^3^

Generally, particulate matter )PM( has different sizes and is divided into coarse particles (PM_10_), fine particles (PM_2.5_) and ultra-fine particles (PM_0.1_).^4^

Epidemiological studies have shown that PM is hazardous for human health,^5,6^ and have reported various adverse effects for PM_10_ including abortion, premature birth, lung cancer, cardiovascular and respiratory disease, and mortality.^7-12^

PM_10_ refers to solid particles often larger than colloid which can be temporarily suspended in air or other gases. Most hurricanes send large amounts of PM_10_ into space, which, in addition to air pollution, can transfer bacteria.^13^ PM_10_ transmitted by air may be chemically neutral or active. They can also absorb matter that are chemically active in the atmosphere and combine with them to form chemically active species. More importantly, these particulate matters can cause chemical damage directly through their corrosive nature. In fact, particulate matter are either intrinsically toxic and interfere with the mechanisms of the respiratory system, or act as carriers of toxic substances.^14^

In recent years, the prevalence of PM_10_ has increased at regional and global levels^15^ In Iran, due to increased dust storms from the neighboring countries, in the west of Iran and environmental and industrial manipulations; PM_10_ has caused various health, economic and environmental problems in the center, west and southwest of Iran and especially in Khuzestan province.^16^ Studies have classified the effects of dust storms into two general groups: environmental and human. Among the environmental impacts are solar radiation blockage^17^ and reduced vision.^18,19^ In human, dust storms cause health issues, such as headache, nausea, sensitivity of the eyes, nose, throat, allergic reactions, asthma, respiratory tract infections,^10,17^ cardiovascular and respiratory diseases, lung cancer and increased mortality .^20^ Other studies show that dust storms have a positive correlation with increased cardiovascular and respiratory case, emergency hospital visits^21-24^ and admissions.^25,26^ In Taiwan, Asian dust storms increased the risk of respiratory disease up to 7.66% one day after the storm, the risk of death on the second day up to 4.92% and 2.59% of the risk of circulatory diseases in the next two days after the storm up to 2.59%.^10^ Meanwhile, some studies did not find a significant relation between increased ambient PM_10_ caused by dust storms and cardiovascular^27,28^ or respiratory diseases mortality.^29^

In the province of Khuzestan (southwestern Iran), traffic accidents, and cardiovascular and respiratory diseases are among the most important medical emergencies. The city of Dezful in Khuzestan province has been exposed to high ambient PM_10_ caused by dust storms for many years; and the emergency medical services (EMS) serve as the first line of emergency treatment.

Although this region has been exposed to severe dust storms in the last decade, there has been few studies on the effect of dust storms on pre-hospital missions in the Middle East. In this study we tried to determine the relation between ambient PM_10_ and the number of daily EMS missions undertaken for cardiovascular or respiratory disease attacks and traffic accidents.

## Materials and Methods

### Study location

**Figure. 1:**
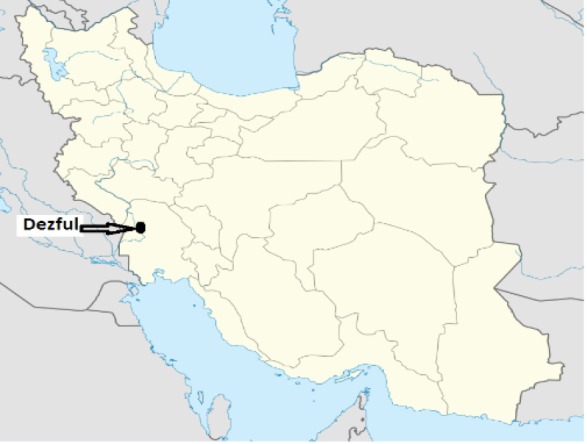
Location of Dezful on the map.

Dezful is a city located in the southwest of Iran (Figure 1). It is the center of the Dezful county, and the thirtieth populated city of the country, with an area of nearly 40 km^2^, located in the plains of Khuzestan province. Dezful is located at 32° 38’ N and 48° 40’ E. According to the 2016 census, 231476 people live in this city.^30^

### Exposure data

Data on the number of pre-hospital cardiovascular, respiratory and traffic accident trauma missions were inquired from emergency medical centers affiliated to Dezful University of Medical Sciences from March 2013 until March 2016, and were classified in age and sex groups.

The emergency medical centers under study supervised 9 active pre-hospital medical stations with on-call physicians, trained technicians, drivers, ambulances and primary care facilities. People from different districts of the city (with different socioeconomic class), contact these centers, in case they need urgent medical attention.

### Exposure assessment

The Khuzestan Province Environmental Protection Agency records the concentrations of PM_10_ in Dezful by one central station on an hourly basis. In this study, values were inquired from March 2013 until 2016 and the average daily amount of PM_10_ was calculated. Other meteorological parameters including maximum, minimum and average daily temperature and relative humidity were obtained from Khuzestan’s Meteorological Organization.

### Analysis

Initially all variables were evaluated for missing data and the Expectation Maximum (EM) method was used to impute missing parameters.^31^

In this time-series study, a generalized linear model (GLM) and a distributed lag model (DLM) were used to investigate the relation between the daily level of PM_10_ and the number of EMS missions due to cardiovascular, respiratory diseases and traffic accident trauma. In the GLM section, the Poisson regression model was chosen because of the enumerated nature of the dependent variable. The important conditions of using this model are the equality of means and variances. In practical cases, if the condition is not met and over-dispersion (where variance is greater than the mean) occurs, the quasi-Poisson model is used. In this study, because of over-dispersion, the quasi-Poisson regression model was used.

Previous research has shown that temperature and humidity may affect cardiovascular and respiratory disease exacerbations and traffic accidents.^2,32^ In order to adjust the effect of temperature and humidity, as confounding variables, a natural cubic spline function with a degree of freedom 6 and 3 was used for temperature and humidity, respectively.^33^ The degree of freedom was determined by minimizing the Akaike’s information criterion (AIC). Similar studies have also used a degree of freedom less than 10.^34^ Because the concentration of PM_10_ may be different on different days of the week and on holidays, the effect of weekdays and holidays were included in the model and their effects were adjusted. The analysis was performed using the R Software (version 3.3.1), and by the analysis of time series and the dlnm package. The significance level in this study was less than 0.05.

## Results

The number of people who visited or contacted EMS centers from March 2013 until March 2016, was 16116, of which the majority were men (11 826), and in the age range of 18 to 60 years old (10 384). Most cases were traffic accident injuries (11369 people). Other details on the number of EMS missions are presented in Table 1.

**Table 1:** The number of EMS missions and descriptive indices of PM_10_ and climate factors in the city Dezful from March 2013 to March 2016

**Variable (per day)**	**N**	**Mean± SD**	**Min**	**Max**	**Q1**	**Median**	**Q3**
Total people	16116	21.2±7.3	1	89	17.0	21.0	25.0
Men	11826	15.6±5.7	0	75	12.0	15.0	19.0
Women	4290	5.6±2.9	0	20	4	5	7
< 18 years	2475	3.3±2.3	0	15	2	3	4
18 to 60 years	10384	13.7±5.1	1	51	10.0	13.0	17.0
> 60 years	2148	2.8±2.0	0	29	1.0	3	4.0
Cardiovascular problems	3176	4.2±2.2	0	17	3.0	4.0	5.0
Respiratory problems	1452	1.9±1.5	0	10	1.0	2.0	3.0
Traffic accident trauma	11369	15.0±6.2	0	62	11.0	14.0	19.0
PM_10_ (μg/m^3^)	-	187.8±289.3	29.4	3421.9	96.1	129.9	187.6
Temperature (°C)	-	27.2±6.9	7.4	41.2	18.3	28.5	37.0
Relative humidity (%)	-	43.5±19.7	13.4	92.6	25.9	39.9	58.8

**Figure. 2:**
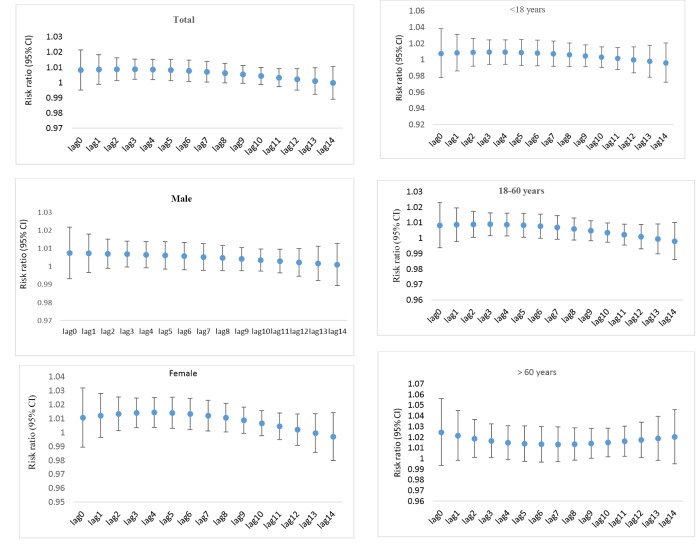
Association between increase in PM_10_ and EMS missions in sex and age-groups up to 14 day lags (in single lag models) in Dezful, Iran, March 2013 – March 2016.

**Figure. 3:**
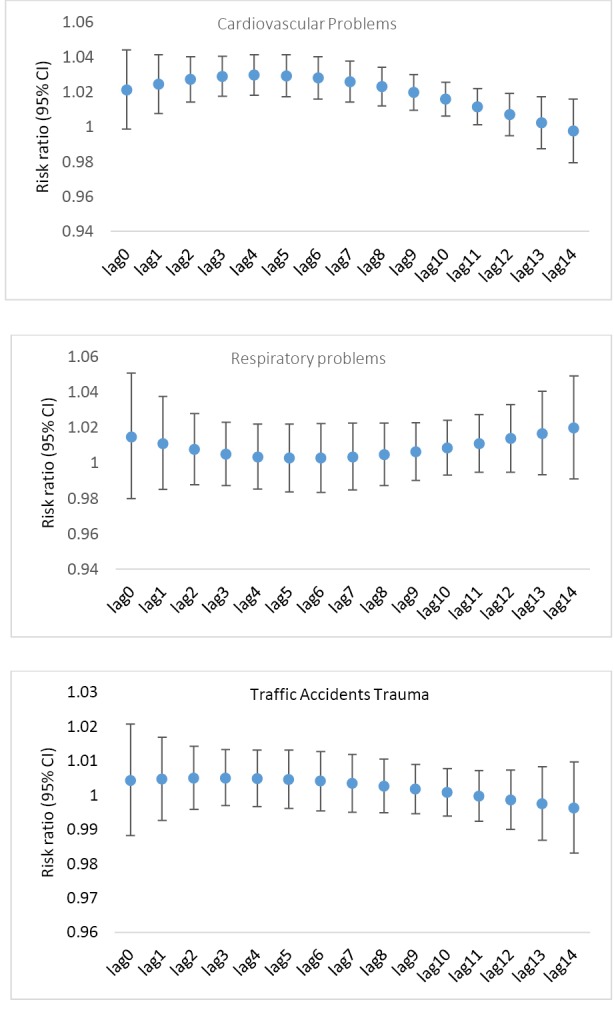
Association between increase in PM_10_ and EMS missions up to 14 days later (in single lag models) in Dezful, Iran, March 2013 – March 2016.

The relation between the number of EMS missions and PM_10_ pollutants in total and in subgroups, per IQR increase in the average daily concentration of PM_10_ (IQR = 91.45 μg/m^3^) in single-lag and accumulative lags are shown in Table 2 and 3 and Figures 2 and 3.

**Table 2:** Relative risk (RR) of EMS missions for each inter-quartile range (IQR) increase in PM_10_ in single and cumulative lag* in Dezful, Iran, March 2013-March 2016, in gender and age subgroups

**Lag**	**Total**	**Male**	**Female**	**<18 years**	**18-60 years**	**> 60 years**
	**RR (95% CI)**	**RR (95% CI)**	**RR (95% CI)**	**RR (95% CI)**	**RR (95% CI)**	**RR (95% CI)**
0- Lag	1.008 (0.995-1.021)	1.007(0.993-1.021)	1.010(0.989-1.031)	1.007(0.977-1.038)	1.008(0.993-1.022)	1.024(0.993-1.056)
1- Lag	1.008(0.998-1.018)	1.007(0.996-1.017)	1.011(0.996-1.027)	1.008(0.986-1.031)	1.008(0.997-1.019)	1.021(0.998-1.044)
2- Lag	1.008(1.001-1.016)^**^	1.007(0.998-1.015)	1.013(1.001-1.025)^**^	1.009(0.992-1.026)	1.008(1.001-1.017)^**^	1.018(1.001-1.036)^**^
3- Lag	1.008(1.002-1.015)^**^	1.006(0.999-1.014)	1.014(1.003-1.024)^**^	1.009(0.994-1.024)	1.008(1.001-1.016)^**^	1.016(1.001-1.032)^**^
4- Lag	1.008 (1.001-1.015)^**^	1.006 (0.999-1.013)	1.014(1.003-1.025)^**^	1.009 (0.994-1.024)	1.008 (1.001-1.0161)^**^	1.014 (0.999-1.030)
5- Lag	1.008 (1.001-1.015)^**^	1.006 (0.998-1.013)	1.013 (1.002-1.025)^**^	1.008(0.993-1.024)	1.008 (1.0005-1.016)^**^	1.013 (0.997-1.030)
6- Lag	1.007 (1.0006-1.014)^**^	1.005 (0.998-1.013)	1.0131 (1.001-1.024)^**^	1.008(0.992-1.024)	1.007 (0.999-1.015)	1.013 (0.996-1.030)
7- Lag	1.006 (1.0001-1.013)^**^	1.005 (0.997-1.012)	1.011 (1.001-1.023)^**^	1.007 (0.991-1.022)	1.006 (0.999-1.014)	1.013 (0.997-1.029)
Lag 0-2	1.025 (0.995-1.055)	1.021 (0.989-1.054)	1.036 (0.988-1.085)	1.025 (0.958-1.096)	1.025 (0.992-1.059)	1.065 (0.994-1.141)
Lag 0-7	1.067 (1.016-1.120)^**^	1.053 (0.998-1.111)	1.107 (1.023-1.198)^**^	1.069 (0.956-1.197)	1.067 (1.011-1.127)^**^	1.144 (1.018-1.285)^**^
Lag0-14	1.090 (1.014-1.171)^**^	1.074 (0.992-1.162)	1.139 (1.014-1.278)^**^	1.079 (0.916-1.271)	1.083 (0.999-1.174)	1.282 (1.081-1.520)^**^

* Adjusted for trend, seasonality, temperature, relative humidity, weekdays and holidays. ** Statistically significant

**Table 3:** Relative risk (RR) of EMS missions for each inter-quartile range (IQR) increase in PM_10_ in single and cumulative lag structure* in Dezful, Iran, March 2013 – March 2016, classified by causes of EMS missions

**Lag**	**Cardiovascular Problems**	**Respiratory problems**	**Traffic Accident Trauma**
	**RR (95% CI)**	**RR (95% CI)**	**RR (95% CI)**
0- Lag	1.0210 (0.998-1.043)	1.014 (0.979-1.050)	1.004 (0.988-1.020)
1- Lag	1.024(1.007-1.041)^**^	1.010 (0.985-1.037)	1.004 (0.992-1.016)
2- Lag	1.027 (1.014-1.040)^**^	1.007 (0.987-1.027)	1.004 (0.995-1.014)
3- Lag	1.028 (1.017-1.040)^**^	1.005 (0.987-1.023)	1.005 (0.996-1.013)
4- Lag	1.029 (1.018-1.041)^**^	1.003 (0.985-1.021)	1.004 (0.996-1.013)
5- Lag	1.029 (1.017-1.041)^**^	1.002 (0.983-1.021)	1.004 (0.996-1.013)
6- Lag	1.027(1.015-1.040)^**^	1.002 (0.983-1.022)	1.004 (0.995-1.012)
7- Lag	1.025 (1.014-1.037)^**^	1.003 (0.984-1.022)	1.003 (0.995-1.011)
Lag 0-2	1.074 (1.022-1.129)^**^	1.0334 (0.955-1.117)	1.014 (0.977-1.051)
Lag 0-7	1.235 (1.136-1.342)^**^	1.0513 (0.920-1.200)	1.036 (0.975-1.101)
Lag0-14	1.333 (1.178-1.508)^**^	1.138 (0.937-1.383)	1.033 (0.945-1.130)

* Adjusted for trend, seasonality, temperature, relative humidity, weekdays and holidays. ** Statistically significant.

The models were adjusted for trend, seasonality, temperature, relative humidity, weekdays and holidays. In the single lags of 2 to 7 days, and cumulative lags of 0-7 and 0-14 days after exposure, for each IQR increase in the average PM_10_ concentration, the relative risk of total EMS missions increased significantly by 0.8, 0.8, 0.8, 0.8, 0.7, 0.6, 6.7, and 1.4 % respectively (Table 2).

In the female population in the adjusted model for 2 to 7 day lags and cumulative lags of 0-7 and 0-14 days after exposure, for each IQR increase in the average concentration of PM_10_, the relative risk increased significantly by 1.4, 1.4, 1.3, 1.3, 1.1, 10.7 and 13.9 % respectively (Table 2).

In the 18-to-60 year old population in the adjusted model for single 2 to 5 day lags, for each IQR increase in the average concentration of PM_10_, the relative risk for the number of EMS missions increased significantly by 0.8% and the relative risk of the cumulative lag of 0-7 days increases by 6.7% (Table 2).

In the population over 60 years old in the adjusted model for 2, and 3 day lags and cumulative lags of 0-7 and 0-14 days, for each IQR increase in the average concentration of PM_10_, the relative risk of EMS missions increased significantly by 1.8, 1.6, 14.4 and 28.2 %, respectively (Table 2). However, the adjusted results in the male population and in the age group under 18 years of age in all single and cumulative lags was not significant (Table 2).

In the adjusted model for single one to seven day lags and cumulative lags of 0-2, 0-7 and 0-14 days, for each IQR increase in the average concentration of PM_10_, the relative risk of EMS cardiovascular missions increased significantly by 2.4, 2.7, 2.8, 2.9, 2.9, 2.7, 2.5, 7.4, 23.5 and 33.3% . But, in all adjusted models, the number of EMS missions due to respiratory problems and traffic accidents was not related to PM_10_ (Table 3).

## Discussion

In recent decades, the increase in ambient particulate matter in the Khuzestan province of Iran has had an adverse effect on the health of the people in this province. The proper management of this crisis requires risk assessment of this phenomenon. This study was conducted, to determine the impact of increased PM_10_ resulting from sand storms on the risk of pre-hospital emergency missions.

The results of this study showed that increase in ambient PM_10_ levels caused by dust storms had a direct and significant effect on emergency pre-hospital missions. Also, the results showed that PM_10_ had a greater impact on sensitive populations including women and the elderly.

A similar study conducted by Sajani et al in Italy, showed that a 10 μg/m^3^ increase in PM_10_ caused a significant increase in the number of non-traumatic emergency ambulance dispatches (0.86%, 95% CI: 0.61-1.1%).^35^ Also, Tasmin et al showed that increase in ambient suspended particulate matter (SPM) increased the risk of emergency pre-hospital ambulances departing for acute illnesses (RR= 1.008 (95% CI:1.007-1.010 )) in Japan.^36^ Ueda et al reported that severe Asian dust storms in Japan increased the risk of EMS missions for all emergency patients (12.1%, 95% CI: 2.3%-22.9%) on the same day and up to 3 days after exposure ^37^

Kwon et al stated that the occurrence of Asian dust storms has a significant relation with cardiovascular and respiratory morbidity and mortality; and one day after the storm the rate of respiratory diseases increased by 6.7%; and two days after the storm overall mortality increased by 4.2% and cardiovascular disease increase by 2.9.^38^

In this study, PM_10_ had a significant impact on elderly people. Maheswaran et al also showed that elderly people in London were more susceptible to the adverse effects of air pollution. The adjusted rate ratios, for each 10 μg/m^3^ increase in PM_10_, for ischemic stroke in all ages was 1.22 (95% CI: 1.77-1.93), for the 40-64 age group was 1.22 (95% CI: 0.55-2.28), and for the 65-79 years group was 1.86 (95% CI:1.10-3.13).^39^ The more severe effects of air pollution on the elderly can be due to homeostatic disorders, changes in immune response, and exacerbation of cardio-respiratory diseases.^40^

This study also showed that increased PM_10_ concentrations was associated with increased risk of pre-hospital emergency missions due to cardiovascular problems. This result is in line with the results of the study by Ebrahimi et al in Sanandej, Iran, which showed that increase in daily ambient PM_10_ concentration, increases the number of cardiovascular and respiratory patients referral to emergency wards (r=0.48, *P* <0.05).^41^ Ueda et al also reported that Asian dust storms increased the risk of EMS missions due to cardiovascular diseases in Japan (20.8%, 95% CI: 3.5% -40.9%), but this increased risk was not observed for respiratory diseases (10.3%, 95% CI: -11.5 - 37.5).^37^ Similar to this study, Tam et al in Hong Kong showed that an increase in PM_10_ increases the number of cardiovascular visits to emergency services (1.02, 95% CI: 1.00-1.05).^22^ Sajani et al in Italy showed that increased ambient particulate matter, increased the risk of cardiovascular (0.44% [95% CI: 0.9120.02] and respiratory missions 0.31% [95% CI: 0.75- 20.13]).^35^

Similar to the results of this study, Barnett et al, showed that the 2009 dust storm in Brisbane, Australia had no significant effect on emergency department visits due to respiratory illnesses.^42^ But, Tasmin et al showed that following increased ambient particulate matter, the rate of ambulance missions due to respiratory diseases significantly increased (1.018, 95% CI: 1.013, 1.023), in Japan; but there was no increase for cardiovascular missions (1.000, 95% CI: 0.996, 1.005).^36^ Differences in the results of different studies are probably due to differences in population susceptibility, the PM_10_ components and the concentration of PM_10_ in dust storms.^28^ As a result, there remains a significant uncertainty in the generalization of risk estimates.

The mechanism that particulate matter affects cardiovascular and respiratory diseases is still controversial. Studies have reported that increase in ambient particulate matter may increase blood viscosity^43^ and plasma fibrinogen^44^ which together predispose individuals to cardiovascular diseases. Also, Nasser et al suggests that this effect may be due to the entrance of particles into the respiratory system, and reduced lung function. Particles that pass through the alveoli and enter the blood circulation, can cause inflammation, and affect the cardiovascular system. On the other hand, in the lungs, particles cause oxidative tension, inflammatory reactions and the release of activated leukocytes and cytokines. These reactions can also increase cardiovascular diseases.^45^

The results of this study did not show a significant relation between increased PM_10_ concentration and EMS missions due to traffic accidents. In this regard, Sajani et al in Italy did not find a significant relation between pre-hospital emergency traumatic missions and increased PM_10_ levels either (0.13, 95% CI: −0.27- 0.54; *P* value 0.739).^35^ The reason might be that because of reduced vision, drivers prefer to postpone their trips until the sand storm clears.

One of the advantages of this time series study was that stable confounders such as age, socioeconomic status and chronic conditions did not need adjustment, because they do not change on a daily basis, and the unit of analysis for this time series study was a day.

### Limitations of study

One of the limitations of this research was that the diagnosis of the patients, was recorded by the emergency medical technician or physician, and this diagnosis might have changed later.

Another limitation of the study is the biases inherent in ecological studies, including aggregated data and the fact that at the results at the aggregated level cannot be generalized directly to the individual level (Ecological Fallacy). In this study, exposure was measured at the population level, not at the individual level. But this was the only way we could measure exposure.

## Conclusion

Overall, the results showed that the increase in PM_10_ caused by dust storms is associated with an increase in the rate of EMS centers’ missions a few days after exposure. Interventions are required to reduce ambient PM levels and exposure to PM, especially for high risk individuals including the women and the elderly.

## Competing interests

The authors declare that they have no conflicts of interest to declare.

## Ethical approval

The study protocol was approved by the Ethics Committee of Dezful University of Medical Sciences (Ethics Code: IR.DUMS.REC.1395.13).

## Funding

This study was funded and supported by Dezful University of Medical Sciences, Grant No:166.

## Acknowledgments

The authors wish to express their gratitude to Mr. Ali Reza Azarian and his colleagues at the Khuzestan Province Environmental Protection Agency for providing data.
